# The effect of clinical experience, judgment task difficulty and time pressure on nurses’ confidence calibration in a high fidelity clinical simulation

**DOI:** 10.1186/1472-6947-12-113

**Published:** 2012-10-03

**Authors:** Huiqin Yang, Carl Thompson, Martin Bland

**Affiliations:** 1 Centre for Reviews and Dissemination, University of York, York,, YO10 5DD, UK; 2 Department of Health Sciences, University of York, York, UK

**Keywords:** High fidelity clinical simulation, Confidence calibration, Clinical experience, Overconfidence, Underconfidence, Time pressure, Clinical judgment, Hard-easy effect

## Abstract

**Background:**

Misplaced or poorly calibrated confidence in healthcare professionals’ judgments compromises the quality of health care. Using higher fidelity clinical simulations to elicit clinicians’ confidence 'calibration' (i.e. overconfidence or underconfidence) in more realistic settings is a promising but underutilized tactic. In this study we examine nurses’ calibration of confidence with judgment accuracy for critical event risk assessment judgments in a high fidelity simulated clinical environment. The study also explores the effects of clinical experience, task difficulty and time pressure on the relationship between confidence and accuracy.

**Methods:**

63 student and 34 experienced nurses made dichotomous risk assessments on 25 scenarios simulated in a high fidelity clinical environment. Each nurse also assigned a score (0–100) reflecting the level of confidence in their judgments. Scenarios were derived from real patient cases and classified as easy or difficult judgment tasks. Nurses made half of their judgments under time pressure. Confidence calibration statistics were calculated and calibration curves generated.

**Results:**

Nurse students were underconfident (mean over/underconfidence score −1.05) and experienced nurses overconfident (mean over/underconfidence score 6.56), P = 0.01. No significant differences in calibration and resolution were found between the two groups (P = 0.80 and P = 0.51, respectively). There was a significant interaction between time pressure and task difficulty on confidence (P = 0.008); time pressure increased confidence in easy cases but reduced confidence in difficult cases. Time pressure had no effect on confidence or accuracy. Judgment task difficulty impacted significantly on nurses’ judgmental accuracy and confidence. A 'hard-easy' effect was observed: nurses were overconfident in difficult judgments and underconfident in easy judgments.

**Conclusion:**

Nurses were poorly calibrated when making risk assessment judgments in a high fidelity simulated setting. Nurses with more experience tended toward overconfidence. Whilst time pressure had little effect on calibration, nurses’ over/underconfidence varied significantly with the degree of task difficulty. More research is required to identify strategies to minimize such cognitive biases.

## Background

The ability of nurses (like all clinicians) to be appropriately confident in their clinical judgments is an important part of safe and effective healthcare. Overconfidence in judgments such as “critical event” (such as cardio pulmonary arrest) risk assessment may result in delayed or inappropriate interventions [1,2]. There is no reason to suspect that nurses are immune to the over/under confidence that afflicts all decision makers [3]. Judgment overconfidence is a particularly important bias [4] in healthcare as overconfident clinicians (erroneously inflating the probability of being correct) are less likely to seek information that could increase the chances of a correct clinical judgment [5]. The relationship between confidence and judgment correctness is known as calibration of confidence [6]. Confidence calibration studies describe the degree of concordance between perceived confidence in an event occurring and the probability of its occurrence. Overconfidence occurs when perceived confidence exceeds judgment correctness. Conversely, underconfidence occurs when judgment performance exceeds perceived confidence.

Overconfidence is a reasoning bias that is not always mediated by clinical experience. Hausman et al. [7] found experienced paediatric residents were more likely to be overconfident; a finding corroborated by Friedman et al. [8] with experienced residents faced with diagnostic judgments. Similar findings have been seen in nurses [9,10].

Confidence in a judgment appears to be linked to the perceived difficulty of a judgment [11-14]. The more difficult the task the greater the overconfidence, and the easier the task the greater the underconfidence [3,15]. This pattern is known as the ‘hard-easy effect’ [6]: participants are underconfident for easy judgments and overconfident for difficult judgments. This ‘hard-easy effect’, particularly overconfidence in difficult judgments, may lead to prematurely ceasing clinical judgment reasoning, resulting in an inappropriate clinical response or action. To the best of our knowledge, the relationship between judgment task difficulty and confidence calibration in clinicians has not been the subject of previous research.

Time is a crucial factor when seeking to understand the relationship between confidence and performance [16]. Judgment confidence tends to increase with the amount of time available for the tasks [17,18], and decrease when judgments are time pressured [19]. Of course, such confidence may be misplaced when performance on a task is examined; too much time spent on a task by an “expert” may induce poorer performance than their average [20]. Nurses faced with assessing critical event risk do so under time pressured conditions. A judgment or decision is made by critical care nurses once every 30 seconds in an average 8 hour shift [21]. So whilst it is reasonable to hypothesise that time pressure will lead to underconfidence in nurses faced with critical event risk judgments, we know very little about the effect of time pressure on confidence calibration performance.

Aside from clinical experience, task difficulty and time constraints, there are significant methodological challenges in seeking to examine the relationship between confidence and judgment performance. One of the most pertinent is the means by which judgments are elicited. Many calibration studies use paper-based scenarios to elicit clinicians’ judgment and confidence ratings. Paper-based simulation is easy to administer but is limited by its lack of face validity, a particularly important limitation for generalising to clinical environments in which clinical information is often perceptual. Using higher fidelity clinical simulation [22] to elicit confidence calibration performance in more “realistic” settings is a tactic that is promising but underutilised. In this study we aimed firstly to explore the potential for using high fidelity clinical simulations to examine nurses’ confidence calibration performance, and then to investigate the effect of clinical experience, task difficulty and time pressure on nurses’ confidence calibration in this realistically simulated situation.

## Methods

### Calibration statistics

A series of calibration statistics were calculated: a calibration score, measures of over/underconfidence and resolution. The calibration score is a weighted squared deviation between the mean proportion of judgments that are correct and the mean confidence rating associated with each confidence category (see equation 1) [3,6].

Equation 1: calibration statistic [12]

(1) 1n∑j=1Jnj(p¯j−e¯j)2

where: (n) represents the total number of responses; (*J*) represents the total number of confidence categories; (*n*_*j*_) represents the number of responses in confidence category (*j);* (${\bar{p}}_{j}$) represents the mean confidence level associated with category (*j);* and (*ē*_*j*_) represents the mean proportion correct in each category (*j).*

Calibration score ranges from 0 (perfect calibration) to 1 (worst calibration). “Perfect” calibration is achieved when the percentage correct is always the same as the confidence level in the judgments assigned in each category by individuals. In contrast, the worst calibration score of 1.0 would be the result of a participant always assigning a confidence score of 100 when their judgments are incorrect, and assigning zero confidence when their judgments are correct.

The over/underconfidence score is an index of the relationship between confidence and accuracy. This score quantifies the deviation between confidence and proportion correct on the basis of the formula: (*p* – *e*)*; where*: (*p*) represents mean confidence rating; and (*e*) represents mean proportion correct. A negative over/underconfidence score denotes underconfidence and a positive score denotes overconfidence.

The resolution score measures a person’s discrimination ability by evaluating how well judges use their confidence ratings to differentiate correct from incorrect responses. Resolution is a weighted squared deviation between the mean proportion correct (*ē*_*j*_) for each confidence category (e.g. 0.50-0.59, 0.60-0.69 and so on) and the overall proportion correct (*ē*) at the whole group level (see equation 2) [12].

Equation 2 Resolution [12]

(2) $$\frac{1}{n}\sum_{j=1}^{J}n_{j}({\bar{e}}_{j}-{\bar{e})}^{2}$$

The resolution score ranges from zero to knowledge index *ē*(1 − *ē*). The resolution score is therefore conditional on the mean proportion correct. This implies that the discrimination skills from two persons cannot be meaningfully compared. A normalised resolution score (NRS) is derived by adjusting for the knowledge index (see equation 3) [23].

Equation 3 Normalised resolution [23]

(3) $$NRS=\left[\frac{1}{n}\sum_{j=1}^{J}n_{j}({\bar{e}}_{j}-{\bar{e})}^{2}\right]/\bar{e}\left(1-\bar{e}\right)$$

The normalised resolution score, which is independent of mean proportion correct, provides a more robust measure when comparing discrimination skills. Normalised resolution scores range from 0 to 1. A higher score is indicative of greater ability to differentiate correct from incorrect responses. The resolution statistic helps further decompose a participant’s judgmental achievement. For instance, if a participant always has a confidence score of 100 on wrong judgments and has zero confidence on correct judgments, the calibration score would be terrible but the resolution score would be perfect. In this paper we report only the results for normalised resolution scores.

### Calibration curve analysis

We constructed calibration curves as another means of examining the relationship between probability judgments and confidence ratings. Each curve is derived by plotting the proportion correct on the y axis against the confidence rating on the x axis [15,24,25]. Plotting a calibration curve requires the conversion of continuous confidence data into ordinal categories (e.g. 0.50-0.59, 0.60-0.69 and so on). The mean proportion correct for each response group is plotted against the corresponding mean confidence rating for that confidence category. A 45 degree line indicates perfect calibration, with deviations away from the 45 degree line indicating the degree of miscalibration (overconfidence and underconfidence). The lower the curve below the perfect calibration line the greater the tendency towards overconfidence. Conversely, the higher the curve above the perfect calibration line the greater the underconfidence.

### The participant sample

We sampled 34 experienced nurses from the population of ward and critical care nurses in North Yorkshire and 63 2^nd^ and 3^rd^ year nurse students from the population of undergraduate students from the University of York, UK. Given the lower recruitment costs associated with student nurses compared to experienced nurses, a ratio of 2:1 for students versus experienced nurse was therefore used for the sample recruitment. Using modestly unequal independent samples such as a ratio of 2:1 can lead to substantial cost saving with only little compromising effect on statistical power [26]. A power analysis was conducted to determine the sample size. This was performed using the variable of judgment correctness for each participant. Our sample gives 90% power to detect a statistically significant difference of approximately 10% in judgment correctness (the correspondence of participants’ judgments with the standard criteria) at 5% significance level (two-sided) between experienced nurses and students.

### The clinical scenarios and judgment criteria

Twenty five scenarios were simulated using a high fidelity mock up of an emergency admission hospital room. Scenarios were generated by randomly sampling patient cases from a dataset of emergency admissions in one NHS District General Hospital during March 2000 [27]. A simulated patient was deemed to be ‘at risk’ if they died, were admitted to Intensive Care or High Dependency Units, or experienced cardiopulmonary resuscitation.

Scenarios were used to simulate the five information cues important for critical event risk assessment: systolic blood pressure, heart rate, respiratory rate, temperature and level of consciousness [28]. All the cues in units were presented in ‘natural units’ (i.e. as they would appear in clinical practice) using a computerised patient simulator (Laerdal ^TM^SimMan, Stavanger, Norway, http://www.laerdal.com) and vital signs monitor. Clinical simulation content was approved by a critical care nurse with ten years of specialist experience as a ward sister in intensive care.

The 25 clinical scenarios were divided into two blocks: scenarios (1–12) and scenarios (13–25). Nurses were placed under time pressure in the first block by allowing only 20 seconds per scenario for a judgment. No time pressure was placed on judgments made in the second block.

### Judgment task difficulty

To investigate whether nurses’ confidence calibration was affected by the ease or difficulty of the clinical judgment task we explored the uncertainty associated with each task. The judgment rule associated with each task was, “if information values are above a clinically significant threshold then classify as ‘at risk’ of a critical event”. We used the Modified Early Warning Scoring system (MEWs) [27] to convert the value of each piece of information into a ‘score’ for each clinical cue (ranging from 0–3). The scores were then summed and the total MEWs score calculated. A total MEWs score of greater than five should, if the rule was being applied, have led to a classification of ‘at risk’. Of course, the uncertain relationship between clinical signs, symptoms and patient outcomes meant that not every scenario (and associated patient case) where the ‘patient’ was classed as ‘at risk’ led to a critical event. Those scenarios in which score and patient outcome were unrelated were classed as difficult, and scenarios in which score was indicative of patient outcome were classed as easier. Classifying scenarios into ‘easy’ or ‘difficult’ ones reflects the complexity of these patient cases in practice. There were 8 difficult scenarios and 17 easier ones.

### Data collection

After being exposed to each scenario in the high fidelity simulation setting participants were asked to make a dichotomous judgment (yes/no: at risk of a critical event) on a data collection sheet (see Additional file 1: Appendix) and assign a level of confidence (0–100) to their judgment. Both experienced nurses and students made 25 dichotomous judgments on risk of acute deterioration on the same 25 simulated scenarios and assigned their confidence ratings for each judgment.

### Ethical approval

Ethical approval for the study was granted by the Health Sciences Research Governance Committee at the University of York, UK. A written informed consent for participation in this study was obtained from each participant.

### Data analysis

Confidence calibration statistics were calculated for each participant. For data appropriate for parametric statistical tests, independent two sample t tests were used to test for the significance of the difference of means in calibration statistics between the two groups. For non parametric data the Wilcoxon rank-sum test was used to test the null hypothesis that the difference of the medians for the calibration indices between the two groups was zero. Analysis of variance (ANOVA) was used to test the mean difference between groups when more than two independent variables were involved. Interactions between independent variables were examined in ANOVA. We used p < 0.05 as a cut off for statistical significance. All analyses were performed using Stata version 9 (http://www.stata.com/).

Confidence curves were plotted. To reduce any bias in the curves, we did not plot data in which confidence was less than 50 (7.51% of confidence ratings). Because of very few data points associated with each confidence category less than 50, deriving a curve based on these very few data points would bias the calibration curve.

## Results

### Participants

Table 1 shows the demographic data of both experienced nurses and student nurses.

**Table 1:** Demographic data of participants

**Demographic characteristics**		**Experienced nurses (n = 34)**	**Student nurses (n = 63)**
Age (years) Mean (SD)		36.55 (9.96)	27.75(8.22)
Clinical experience (years) Mean (SD)		12.15 (9.90)	n/a
Gender n/(%)	Male	5 (15%)	7(11.1%)
	Female	28 (85%)	56(88.9%)

n/a: not applicable.

#### Proportion correct

In the high fidelity clinical simulation settings, no significant differences in proportion correct were found between the student (mean 73.7%; SD 6.88%) and experienced nurse group (mean 73.5%; SD 9.08%), t (95) = 0.11, P = 0.91. The ease or difficulty of the scenario exerted a statistically significant effect (F (1, 289) =247.76, P < 0.001) on the proportion of correct judgments. Time pressure did not significantly alter the number of correct judgments (F (1, 289) =0.00, P = 0.97). Similarly, the proportion correct did not significantly vary between each participant (F (96, 289) =0.74, P = 0.96). No significant interaction was observed between time pressure and easy/difficulty of the scenarios on proportion correct (P = 0.59).

### Confidence ratings

Experienced nurses (mean 80.09; SD 10.47) were significantly more confident than students (mean 72.66; SD10.74), t (95) = −3.28, P = 0.001. Participants’ confidence ratings varied significantly as a result of the difficulty of the cases (F (1, 289) =133.94, P < 0.001) and between participants (F (96, 289) = 7.70, P < 0.001).

Whilst time pressure had no significant effect on confidence on its own (F (1, 289) = 1.78, P =0.18), there was a significant interaction (P = 0.008) between time pressure and the relative difficulty of the task on confidence. Clearly, time pressure had different effects on confidence for easy and difficult scenarios: time pressure increased participants’ confidence for easy judgments whilst time pressure decreased their confidence for difficult cases.

### Under/overconfidence

Students were underconfident (mean over/underconfidence score −1.05; SD 13.41) and experienced nurses were overconfident (mean over/underconfidence score 6.56; SD 15.68), t (95) = −2.51, P = 0.01.

### Calibration & resolution

Experienced nurses were no better calibrated (median 0.048) than students (median 0.048), z = −0.25, P = 0.80. Similarly, students (median 0.198) and experienced nurses (median 0.192) did not differ significantly in their ability to discriminate between their correct and incorrect judgments, z = 0.67, P = 0.51.

### Calibration curve analysis

**Figure 1:**
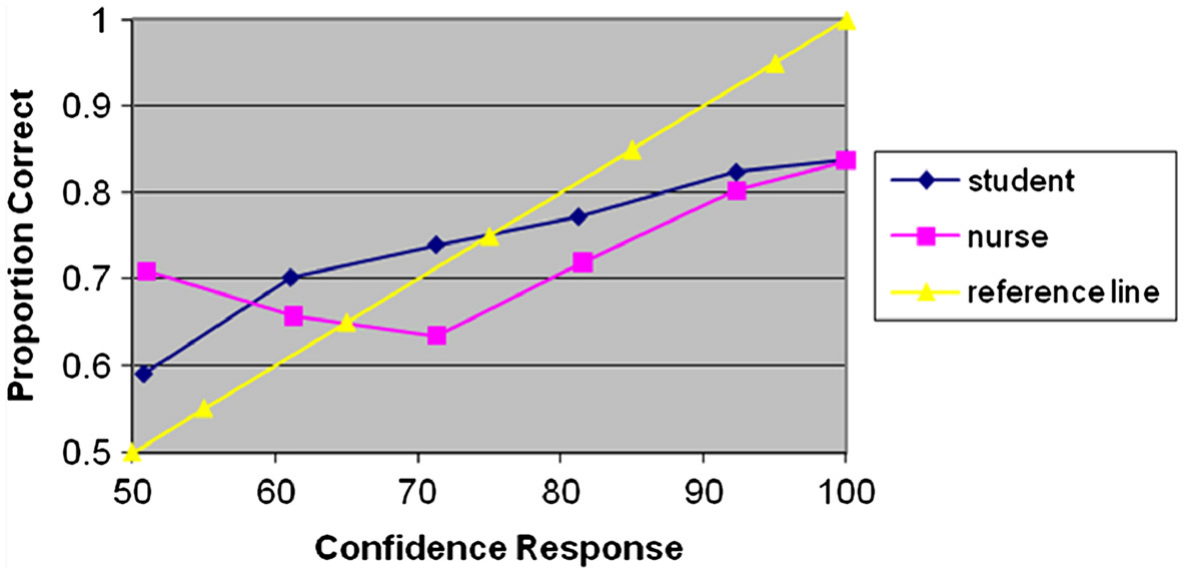
Calibration curves for student and experienced nurses.

Figure 1 shows the calibration curves for students and experienced nurses in the high fidelity simulated situation. Both groups tended toward assigning confidence ratings that were too extreme; a pattern labelled “over-extremity” [24].

**Figure 2:**
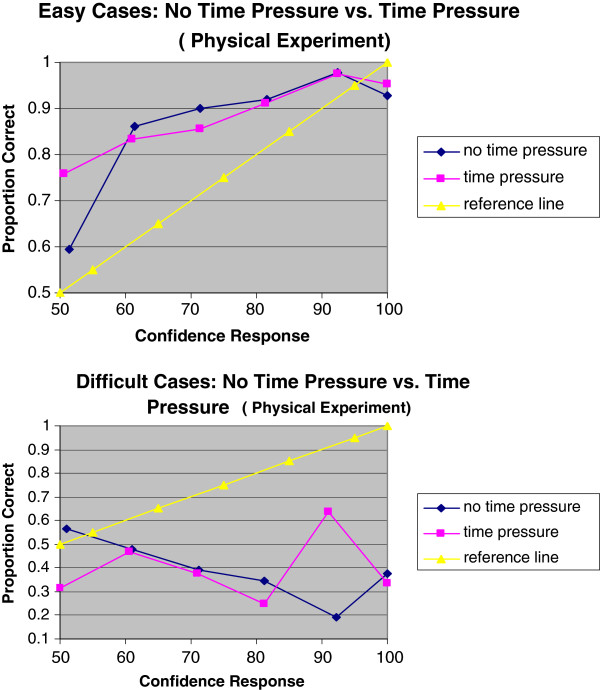
Calibration curves of no time pressure and time pressure for easy/difficult cases.

**Figure 3:**
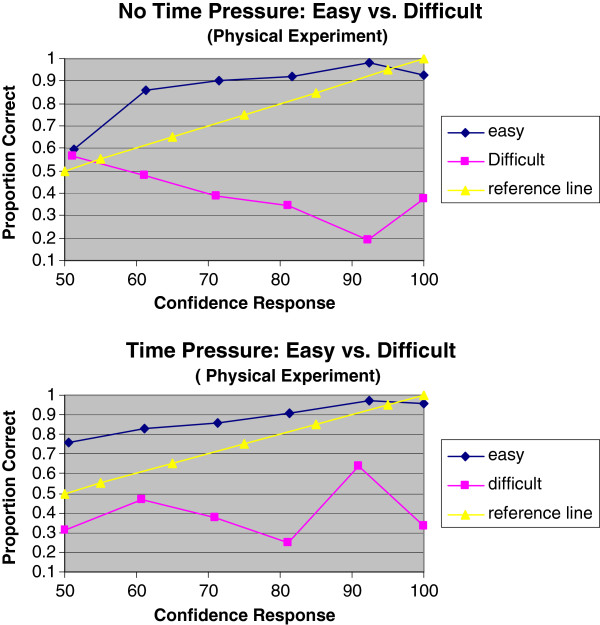
Calibration curves of easy and difficult judgments under no time pressure/time pressure.

Figure 2 shows that time pressure had little effect on participant calibration judgment. Figure 3 shows the calibration curves of easy and difficult scenarios on no time pressure and time pressure, indicating that the hard-easy effect was a distinctive phenomenon in the high fidelity simulated situation.

## Discussion

In capturing and reporting the less than optimal confidence calibration of nurses and students, this study offers both information (and a methodology) for those developing high fidelity clinical simulations (particularly for assessment of critical care events). In the high fidelity clinical simulation environment, we observed a clear relationship between nurses’ subjective confidence ratings and accuracy in their risk assessments: experienced nurses were generally overconfident, while student nurses tended toward underconfidence. The difference of this measure between the two groups was statistically significant (p = 0.01). The findings showed that the subjective probability judgments of experience nurses and students were subject to systematic bias; either they overestimated or underestimated their judgmental abilities or knowledge of self judgment. Our findings replicate the more general psychological picture that suggests people (including decision makers with more experience) are often systematically overconfident with regard to judgment accuracy [6,24,29-34].

An appropriate level of confidence, given someone’s clinical experience, is one marker of a nurse’s competency, and clinical experience is a significant factor in building confidence in nurses’ judgment [9,35,36]. Our study showed that experienced nurses were significantly more confident in their judgments than students, and that nurses’ confidence increased in line with clinical experience. However, we saw no significant benefit on judgment accuracy arising from clinical experience on judgment accuracy in the high fidelity clinical simulation environment. Similar findings were also observed in other studies. For instance, the study by Oskamp [37] showed that experienced clinicians’ judgments were no better than those of graduate students. A further study by Corcoran [38] did not find better performance in the accuracy of treatment plans developed by experienced nurses compared with novice nurses. Hamers et al. [39] also observed a similar level of assessment performance in pain intensity between experienced and student nurses. Ericsson et al. [40] demonstrated that a failure to reliably isolate superior performance amongst nurses with extensive years of experience appears to be a common trend. A similar pattern was observed in doctors; the systematic review of effects of clinical experience on medical performance showed a higher risk of providing lower quality of care in doctors with more years of clinical experience [41].

Given that nurses experience significant amounts of audio and visual information (which is a mix of important signals and ‘noise’) in daily clinical activities, it is reasonable to hypothesise that experienced nurses are more likely to have better calibration performance than student nurses in high fidelity simulated conditions. However, our findings showed no significant difference in calibration and resolution between experienced and student nurses. Our study does not support the hypothesis that confidence calibration performance is a linear function of clinical experience, even in the less than perfect environment of the high fidelity clinical simulation.

### Task difficulty and calibration

Our findings further reveal that nurses’ calibration differs with the difficulty of the judgment task they are faced with. Nurses’ calibration and resolution were generally worse on the more difficult and uncertain tasks. By varying the task difficulty, a hard-easy effect was seen: nurses are overconfident in hard judgments and underconfident in easy judgments.

Similar findings have been documented in psychological studies since the 1970s [3,11-14]. These studies consistently conclude that the extent of miscalibration relies on the degree of ease or difficulty of tasks: overconfidence is most extreme in judges faced with tasks of greater difficulty [6]. Lichtenstein et al. [6] note that the hard-easy effect seems to arise from people’s inability to appreciate the ease or difficulty of a task. Therefore, nurses’ confidence miscalibration may reflect a lack of sensitivity (and commensurate lack of subjective probability adjustment) to the difficulty of tasks.

As with confidence miscalibration, nurses’ ability to resolve information altered as a result of task difficulty: their ability to differentiate correct from incorrect judgments decreased as task difficulty increased. This is also consistent with the psychological literature [42-44] showing that resolution is often better in easier judgments. Similarly, the nurses’ discrimination abilities differed drastically between easy and difficult tasks; with discrimination fairly good on easy tasks, but deteriorating as tasks become more difficult. The strikingly different calibration curves for the two levels of task difficulty (Figure 3a and 3b) may result from nurses’ not really “knowing” their judgments, particularly in difficult cases.

### Time pressure and calibration

Time pressure had no significant impact on nurses’ confidence, the percentage of correct judgments or their overall calibration. This finding runs counter to those studies [17-19], showing that decision makers’ confidence lessens under time pressure and tends to increase with the amount of time spent on tasks. One plausible explanation for this finding is that nurses may experience a “mild” state of time pressure that does not necessarily reduce their confidence. Thus, without sacrificing confidence and accuracy, nurses adapt well to this state of time pressure by accelerating information processing under time constraints. This is in line with the thesis that humans think “adaptively” in situations in which resources are limited [20].

Time pressure increased nurses’ confidence in easy cases and reduced nurses’ confidence in the difficult ones. Such a significant interaction revealed that time pressure had a different effect on confidence between easy and difficult judgments. This phenomenon could be partially explained by “the need for closure” effect [45]. Need for closure refers to a need for certainty, it arises from the impact of time pressure on participants’ motivation and confidence [46-48]. Particularly, when an immediate judgment must be made within a limited time, the need for closure motivates participants to consider fewer hypotheses and be more confident in their favoured hypothesis. Thus, the raised confidence is highly correlated with the (perceived) need for closure. In contrast, without the need for closure (i.e. no time pressure), nurses would tend to seek more information in information processing with a number of competing hypotheses considered, thereby reducing their confidence in any hypothesis. These studies, however, did not differentiate the effect of need for closure on easy and difficult cases. In this study, the need for closure under time pressure significantly impacts on easy judgments in the form of increased confidence. However, the inverse effect of time pressure on confidence associated with difficult tasks suggests that it acts differently in difficult judgment situations.

Recent evidence has shown a significant interaction between the need for closure, judgmental performance and changing judgment task demands (for example, altered task difficulty) [46]. Roets et al. [46] suggest that once tasks are perceived as difficult, willingness to invest effort is reduced, even though the task demands are high with an initial high level of motivation arising from the need for closure. The feeling of investing a great deal of cognitive effort in difficult tasks in a judgment process can decrease the level of confidence [49,50]. Furthermore, others have shown that task difficulty has a significant influence on judgmental response times: response time increases as judgment difficulty increases [12]. Thus response time is often required to be longer in difficult judgments than easy judgments. Our findings suggest that, due to minimising cognitive efforts for difficult judgments under time pressure, it is reasonable that nurses correspondingly assign lower confidence to difficult judgments that require more cognitive efforts when the response time is decreased.

### Limitations

A non-random sampling method to enrol nurse participants was a limiting feature of the study. Whilst deliberately sampling experienced and inexperienced nurses allowed us to investigate the mediating effect of clinical experience on confidence calibration, we could have increased the risk of non-representativeness within subgroup samples. Furthermore, the focus on judgment task of risk assessments in acute care means that the generalisation of the findings to other settings is limited. Further research is required to establish whether the patterns of confidence miscalibration observed in this study are replicated in different clinical contexts.

## Conclusions

Nurses were miscalibrated when matching judgment confidence to judgment performance in a high fidelity simulated environment. Simply being clinically experienced did not help: it just increased the probability of being overconfident. The study revealed that time pressure is an important influence in nurses’ judgments; as time pressure increased – for easier cases so did nurses’ confidence. However increased time pressure led to reduced nurses’ confidence in difficult cases. While time pressure had little effect on nurses’ overall calibration, the observed ‘hard-easy effect’ suggests that nurses’ confidence miscalibration is contingent on task difficulty. These findings highlight the need for nurses to recognise the ‘uncertainty’ [51] associated with clinical judgments they face if their clinical judgments are to be as good as possible. Of course, describing limitations is only the first step in designing effective interventions for minimising nurses’ cognitive biases – interventions that as yet are largely unevaluated.

## Competing interests

The authors declare that they have no competing interests.

## Authors’ contributions

HY and CT were responsible for the study conception and design. HY performed the data collection. HY and MB performed the data analysis. HY was responsible for the drafting the manuscript. HY, CT and MB made critical revisions to the paper for important intellectual content. HY and MB provided statistical expertise. All authors read and approved the final manuscript.

## Pre-publication history

The pre-publication history for this paper can be accessed here:

http://www.biomedcentral.com/1472-6947/12/113/prepub

## Supplementary Material

Additional file 1 **Appendix.** High fidelity clinical simulation scenario. Click here for file
