# Supplementary material for: The effect of clinical experience, judgment task difficulty and time pressure on nurses’ confidence calibration in a high fidelity clinical simulation
Source: BMC Med Inform Decis Mak. 2012 Oct 3;12:113. doi: 10.1186/1472-6947-12-113 (PMC3547784; doi:10.1186/1472-6947-12-113)
Supplement: Additional file 1 — Appendix. High fidelity clinical simulation scenario. [file 1472-6947-12-113-S1.doc]

A**ppendix 1: High fidelity clinical simulation scenario**

***Clinical background Information***

Mr. Robert Wright, 63 years old and 76 kg weight, was presented to the emergency room in your hospital, accompanied by his wife. He was generally feeling unwell, with a tender abdomen and vomited after each meal for past 2 days. He was born in England and he has been married for 38 years. He is a senior engineer in an automotive company. He has no food or medical allergies. There was no report of use of medications. He has no significant past medical history or history of mental illness. The details of family history are unclear. The sets of clinical information relating to the patient’s vital signs from the computerised patient simulator (Laerdal ™SimMan) and vital signs monitor are available to you when you assess Mr. Wright on admission. Please make your judgments for each scenario.

An example of case scenario information simulated by the computerised patient simulator

(Laerdal ™SimMan)

| Systolic blood pressure 134 mmHg  Heart rate 94 beats per minute  Respiratory rate 16 breaths per minute  Temperature 36.8 °C  Conscious level Alert |
| --- |

An example of the response sheet for the high fidelity simulated patient case

| Frame (Scenario) 3 | Risk (circle)  YES NO  Confidence (0-100) |
| --- | --- |
